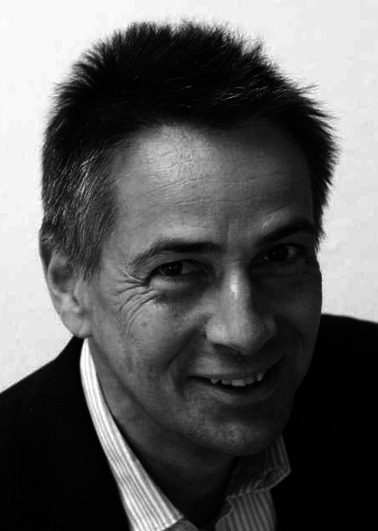# Catalytic Generation of Carbanions through Carbonyl Umpolung

**DOI:** 10.1002/anie.202105469

**Published:** 2021-06-30

**Authors:** Shun Wang, Burkhard König

**Affiliations:** ^1^ Faculty of Chemistry and Pharmacy University of Regensburg Universitaetsstrasse 31 93053 Regensburg Germany

**Keywords:** carbanions, photocatalysis, Umpolung, Wolff–Kishner reaction

## Abstract

Carbonyl Umpolung is a powerful strategy in organic chemistry to construct complex molecules. Over the last few years, versatile catalytic approaches for the generation of acyl anion equivalents from carbonyl compounds have been developed, but methods to obtain alkyl carbanions from carbonyl compounds in a catalytic fashion are still at an early stage. This Minireview summarizes recent progress in the generation of alkyl carbanions through catalytic carbonyl Umpolung. Two different catalytic approaches can be utilized to enable the generation of alkyl carbanions from carbonyl compounds: the catalytic Wolff–Kishner reaction and the catalytic single‐electron reduction of carbonyl compounds and imines. We discuss the reaction scope, mechanistic insights, and synthetic applications of the methods as well as potential future developments.

## Graphical abstract

Novel catalytic strategies for the generation of functionalized carbanionic species by carbonyl Umpolung are discussed in this Minireview. The application of metal catalysis and photocatalysis significantly facilitates the generation and conversion of carbanionic intermediates starting from carbonyl compounds.

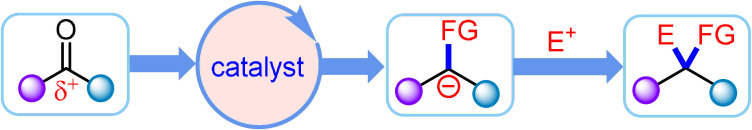

## 1 Introduction

The concept of Umpolung, first introduced by Wittig^[1]^ and then popularized by Seebach, describes the polarity inversion of a particular functional group in organic chemistry.^[2]^ Umpolung reactions create new reactivity by inverting the natural polarity of common organic functionalities and consequently enable the development of unconventional bond‐forming strategies in organic synthesis. With Umpolung tactics, retrosynthetic disconnections can be performed in an inverted manner, permitting the assembly of a target molecule from synthons with both normal and reversed polarities. Over the past few decades, the successful development of numerous C−C bond‐forming Umpolung reactions has greatly expanded the repertoire of methods for organic synthesis.

**Scheme 1:**
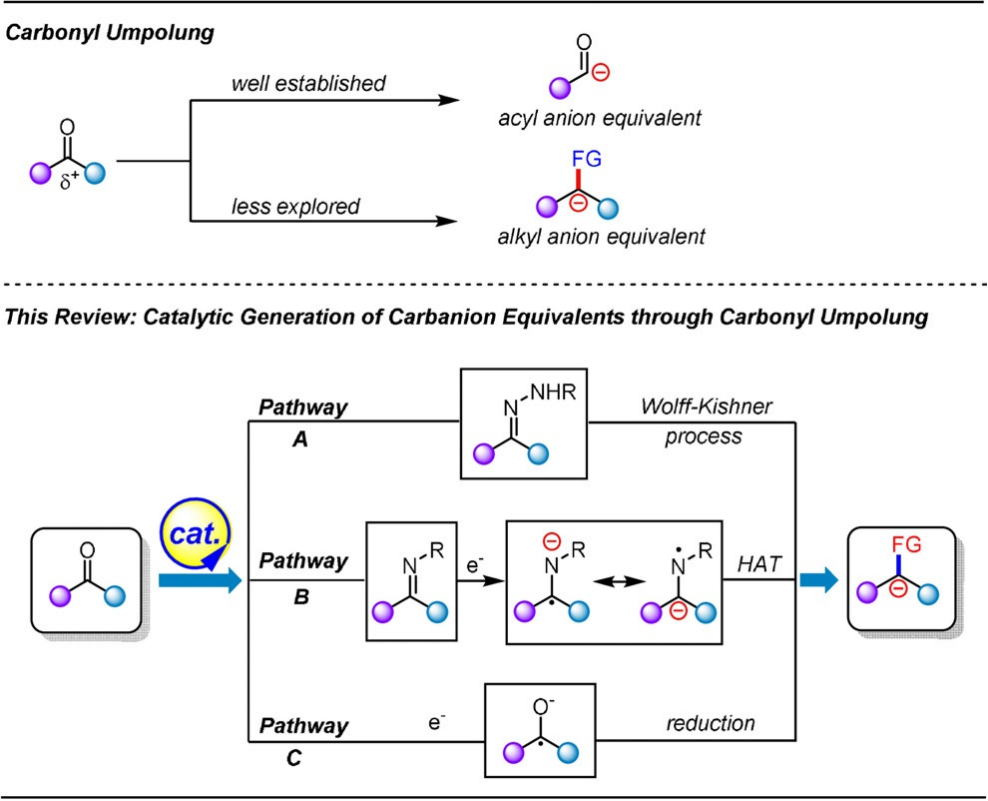
Catalytic generation of carbanions by carbonyl Umpolung.

In the case of the carbonyl (C=O) functionality, the electronegativity of the oxygen atom renders the carbon atom of the carbonyl group partially positively charged, which can be attacked by nucleophilic species. In a typical carbonyl Umpolung, the electronic nature of the carbonyl carbon atom is inverted from electrophilic to nucleophilic. This allows chemists to construct new chemical bonds by reacting the newly obtained carbon nucleophiles with various electrophiles. Two types of synthetically valuable anionic synthons are obtained through carbonyl Umpolung: acyl anion equivalents and alkyl carbanionic intermediates (Scheme 1).

Research interest in the generation of acyl anion synthons by carbonyl Umpolung dates back to the cyanide‐catalyzed benzoin reaction reported in 1832.^[3]^ However, systematic and more vigorous Umpolung research started with Corey and Seebach, who described the use of dithianes to access acyl anion synthetic equivalents from aldehydes in the 1960s.^[4]^ The development of N‐heterocyclic carbene (NHC) catalysis provides another valuable and important addition to the chemistry of acyl anion equivalents, with great efforts devoted to the design and synthesis of novel NHC catalysts that catalyze the carbonyl Umpolung reactions.^[5]^ The three above‐mentioned Umpolung strategies constitute the most widely used approaches to access acyl anion species from carbonyl compounds. These seminal discoveries mark true breakthroughs in synthetic method development in the past decades and they have stimulated significant activity in designing efficient catalysts for carbonyl Umpolung.

On the other hand, Umpolung strategies can be applied for the generation of alkyl carbanionic species from a C=O moiety. In comparison to its well‐explored acyl anion counterpart, the generation of alkyl carbanions by carbonyl Umpolung has received considerably less attention. This type of carbonyl Umpolung holds great synthetic potential considering that the generated alkyl carbanion is a versatile intermediate for the construction of C−C bonds on reacting with electrophiles. The earliest example of this Umpolung dates back to the Wolff–Kishner (WK) reduction, in which carbonyl functionalities are converted into methylene groups.^[6]^ After more than one century since its discovery, the WK reaction and related modified reactions remain an important carbonyl deoxygenation method in modern synthesis.[^6a^, ^7^] Recent developments have provided new routes for the catalytic generation of carbanionic intermediates through the merger of the WK process and other catalytic reaction systems, such as transition‐metal catalysis and photoredox catalysis. These advances have greatly expanded the application of this century‐old reaction and, therefore, have received increasing research interest.

Furthermore, in recent years, new techniques for the catalytic generation of carbanionic species through the single‐electron reduction of imines and carbonyl compounds have emerged. Classically, the reduction of imines or carbonyl compounds requires the use of stoichiometric reactants (e.g. low‐valent metals)^[8]^ or electrochemical methods^[9]^ for the reduction. Recent advances have demonstrated that photocatalysis can not only serve as an ideal replacement for the classic reaction system for the C=O/N moiety reduction, but also generate new carbanionic reactivity from carbonyl Umpolung.[^10^, ^11^]

We summarize and discuss in the following sections the catalytic generation of alkyl carbanionic species from carbonyl Umpolung, particularly emphasizing emerging new technologies and trends. We structure our discussion along the two major reaction pathways for the generation of alkyl carbanions from carbonyl compounds: the catalytic WK process (Scheme 1, pathway A) and the catalytic single‐electron reduction of imines and carbonyl compounds (Scheme 1, pathways B and C).

## 2 Catalytic Methods for the Generation of Alkyl Carbanions from Carbonyl Compounds

### 2.1 Catalytic Wolff–Kishner Reaction—Pathway A

**Scheme 2:**
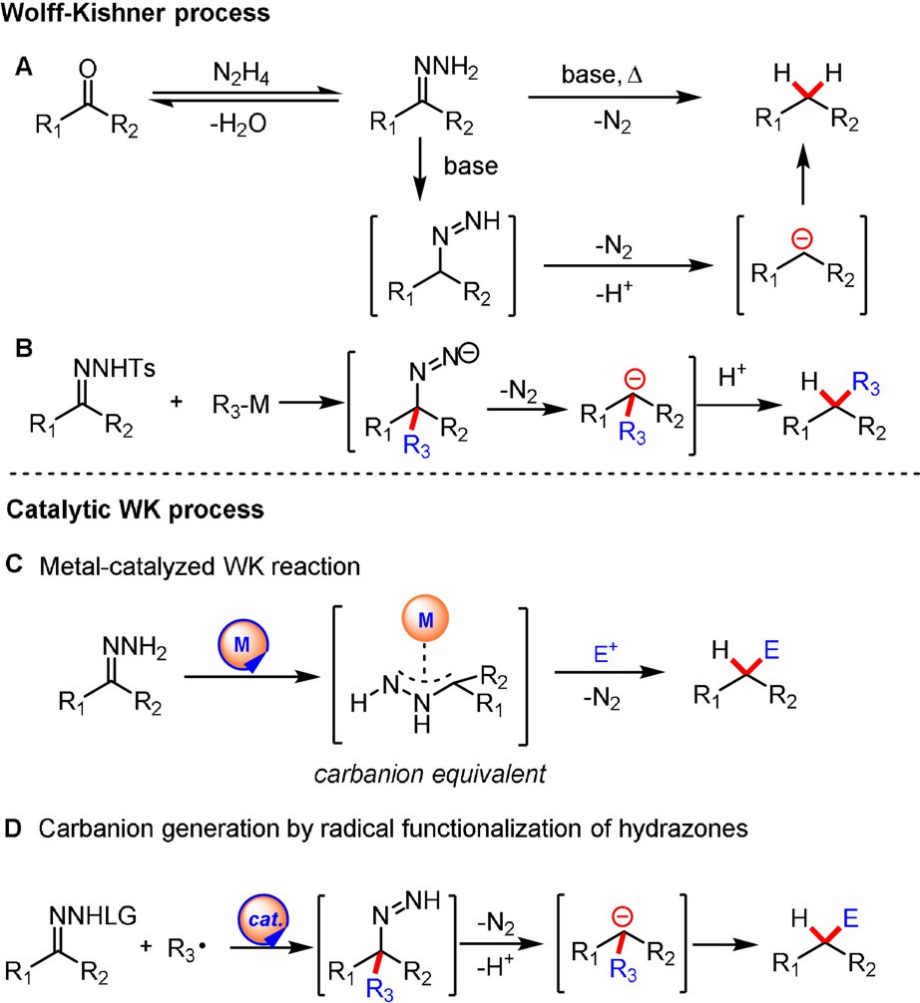
Generation of carbanions by catalytic WK processes.

Before discussing the catalytic WK process, it is worthwhile to first give an overview of the WK reaction mechanism. In a regular WK process, the reaction proceeds through the formation of hydrazones from a carbonyl compound and hydrazine. A subsequent deprotonation‐tautomerization sequence occurs to give the diazene intermediate from the hydrazone. After a second deprotonation, this intermediate rapidly loses molecular nitrogen to give the carbanion, which is then protonated to yield the alkane product (Scheme 2 A).^[6a]^ The formation of the two key intermediates—a hydrazone and the diazene anion—can considerably influence the course of a WK process. Many elegant modifications, including the well‐known Huang‐Minlong,^[12]^ Barton,^[13]^ Cram,^[14]^ and Henbest^[15]^ procedures, have focused on a more efficient formation of the hydrazone by removal of water from the reaction medium, whereas Caglioti^[16]^ and Meyers^[17]^ have utilized substituted hydrazones as alternatives to the classical WK reaction, thereby allowing the facile generation of the diazene intermediate. In particular, the use of sulfonyl hydrazone in the Caglioti modification not only allows the WK reduction to occur under mild conditions but also inspired the generation of substituted carbanions by reacting sulfonyl hydrazone with organometallic reagents (e.g. RLi, RMgBr; Scheme 2 B).^[18]^ This reaction pathway represents one of the earliest pathways for the utilization of the WK process for construction of a C−C bond.

Despite being a very effective way of producing carbanions, synthetic applications of the WK reaction have been mainly limited to carbonyl deoxygenation.^[6a]^ Only a few attempts have been made over the past few years to develop new catalytic systems, namely catalytic variants of the WK reaction for the generation and/or trapping of the generated alkyl carbanions, thus forging C−C bonds. However, the transformation bears the potential for a plethora of carbanion‐based transformations by merging the WK process with other catalytic systems (e.g. transition‐metal catalysis, photoredox catalysis; Scheme 2 C,D) that wait to be further discovered.

#### 2.1.1 Metal‐Assisted Trapping of Carbanions

**Scheme 3:**
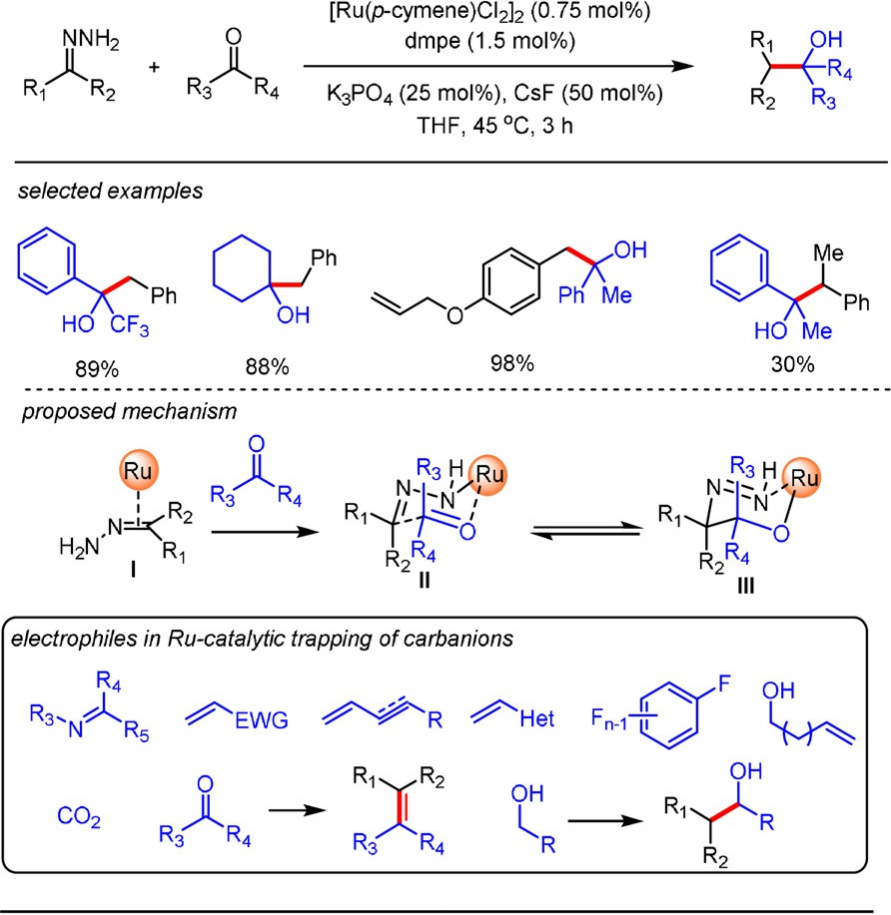
Ru‐catalyzed trapping of carbanionic species generated in the WK process.

The Li group made significant progress in the catalytic trapping of the carbanion in a WK process. In 2017, they first described a ruthenium‐catalyzed strategy for the facile capture of the carbanionic species generated in a WK process from carbonyl compounds (ketone and aldehydes) to give the desired Grignard‐type products.^[19]^ The reaction demonstrates broad functional‐group tolerance for both the hydrazone and carbonyl compounds with different substitution patterns. Notably, the reaction system is compatible with functional groups (including ester, amide, as well as tertiary alcohols), which are normally incompatible with classical Grignard reactions. In their mechanistic proposal, ruthenium complex **I** was initially generated from the association of hydrazone with the ruthenium catalyst. The resulting Ru complex **I** interacts with carbonyl compounds via a Zimmerman–Traxler transition state **II** followed by its rearrangement to give intermediate **III**. The Grignard‐type addition product was released from **III**, concomitant with regeneration of the Ru catalyst. The coordination of the ruthenium complex with the substrate is believed to promote the decomposition of hydrazine,^[20]^ thereby allowing N_2_ extrusion from hydrazone under mild conditions. This new catalysis opens new possibilities to harness the carbanions generated from readily available carbonyl compounds in the WK reaction for catalytic C−C bond‐forming reactions. The Ru catalytic system was later extended and the carbanionic species reacted with a broad range of electrophiles, including imines,^[21]^ alkenes,[^22^, ^23^] CO_2_,^[24]^ dienes,^[25]^ multi‐fluoro arenes,^[26]^ carbonyl compounds (for C=C bond construction),^[27]^ and in situ generated carbonyl compounds (Scheme 3).^[28]^

**Scheme 4:**
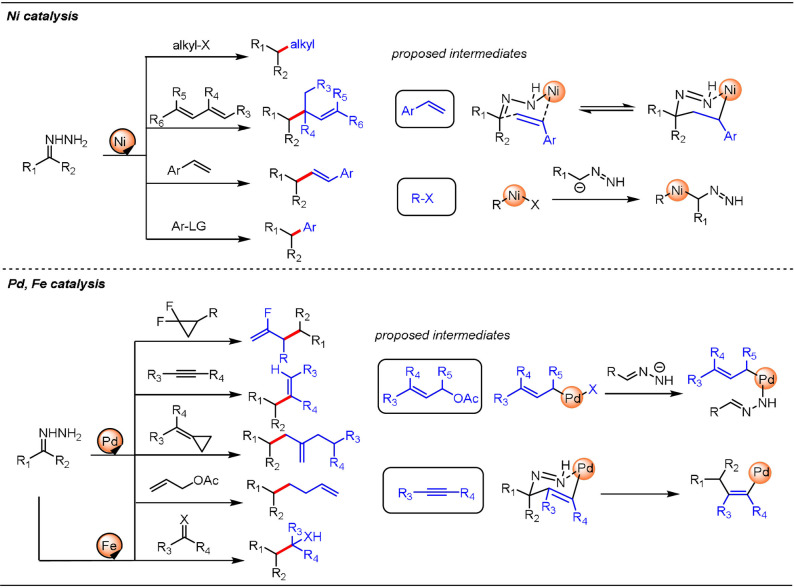
Metal‐catalyzed trapping of carbanionic species generated in the WK process.

In addition, the Li group and others have demonstrated that other transition‐metal catalysts, including Ni, Pd, and Fe,^[29]^ could efficiently be merged with the WK processes to realize different mechanistic pathways (Scheme 4). This results in a vastly expanded set of accessible coupling partners, such as alkyl halides,^[30]^ dienes,^[31]^ styrenes,^[32]^ phenol derivatives,^[33]^ and aryl halides^[34]^ in Ni catalysis and methylenecyclopropanes,^[35]^ alkynes,^[36]^ allylic acetates,^[37]^ and *gem*‐difluorocyclopropanes^[38]^ in Pd catalysis. Several plausible reaction pathways for the different catalytic systems were postulated, as shown in Scheme 4. When styrenes and alkynes were employed as coupling partners, the authors proposed six‐membered intermediates, analogous to the Zimmerman–Traxler transition state proposed for the Ru‐catalyzed Grignard‐type reaction, as the key species for the construction of new bonds. Ligand exchange between the deprotonated hydrazone (C‐nucleophile or N‐nucleophile) and the metal complex (Ni or Pd) was proposed as the key step in the catalytic cycle in the case when (pseudo)halides were used as electrophiles. Combining transition‐metal catalysis with the WK reaction creates unprecedented reactivity for widespread use in organic synthesis.

#### 2.1.2 Carbanion Generation by Radical Addition to Sulfonyl Hydrazones

Nucleophilic addition of organometallic reagents to C=N bonds of sulfonyl hydrazones leads to the generation of substituted carbanions (Scheme 2 B).[^18^, ^39^] However, this approach has a limited functional‐group compatibility, and competing side reactions may occur. Moreover, the number of nucleophiles that can be engaged in such a modified WK process is limited. Aiming to overcome these limitations, strategies for the addition of radical species to sulfonyl hydrazone derivatives were developed.

**Scheme 5:**
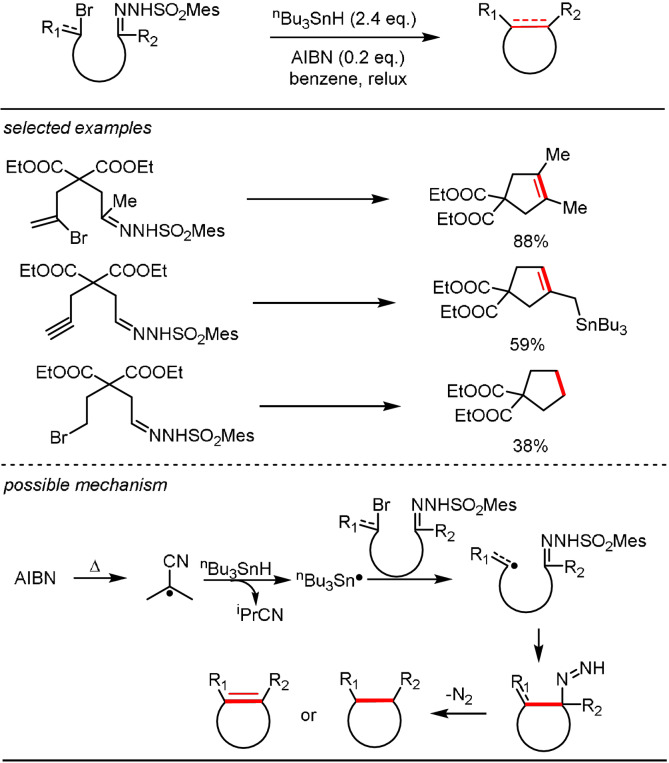
Intramolecular radical functionalization of sulfonylhydrazones.

The earliest example which invokes such a radical‐initiated WK reaction pathway was reported by Kim and Cho as early as 1992.^[40]^ In their report, the intramolecular addition of alkyl or vinyl radicals, generated from alkyl or vinyl bromides under AIBN/^n^BuSnH conditions, to a C=N bond affords a cyclized diazene intermediate. The alkyl diazene forms a cyclized alkane as product, likely via a carbanion intermediate, whereas its allylic diazene counterpart undergoes a pericyclic rearrangement to give an endocyclic alkene product after N_2_ extrusion (Scheme 5).

**Scheme 6:**
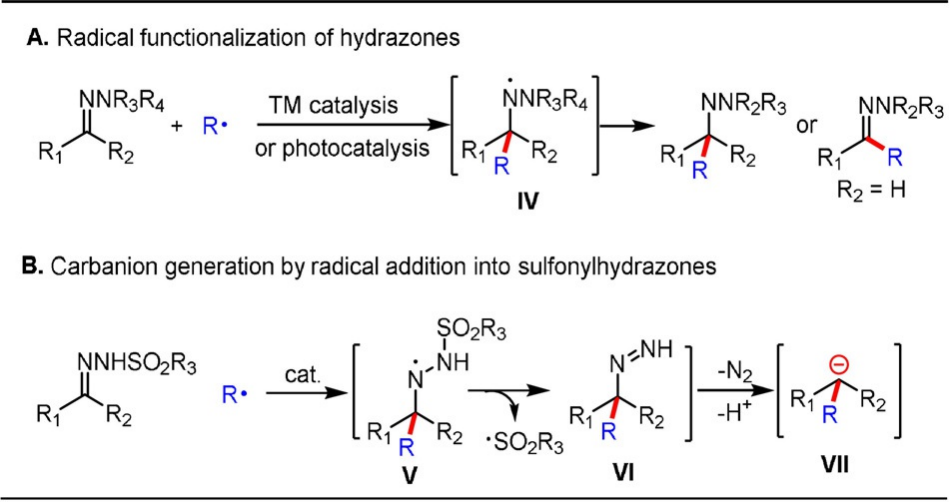
Catalytic radical functionalization of hydrazones.

Although this reaction could be deemed as a representative radical‐initiated WK process for the versatile formation of diazene species, this chemistry has been little explored over the past few decades, with only a few scattered examples reported.^[41]^ However, this reaction sequence needs to be revisited in view of reports of radical addition to other hydrazone acceptors (e.g. *N*,*N*‐dialkyl hydrazones) under transition‐metal or photoredox catalysis (Scheme 6 A).[^42^, ^43^]

In a catalytic radical functionalization approach, aminyl radical intermediate **V** is first generated from radical addition to the C=N moiety of an *N*‐sulfonyl hydrazone (Scheme 6 B). The resulting N‐centered radical undergoes β‐fragmentation to release a sulfonyl radical, thereby affording the diazene intermediate **VI**.^[40]^ This diazene then enters a WK reaction sequence to afford the desired carbanion **VII** after N_2_ extrusion. There are several distinct advantages in introducing new catalysis to this reaction sequence: milder reaction conditions to access carbanion equivalents and the facile generation of functionalized carbanions using different radicals.

**Scheme 7:**
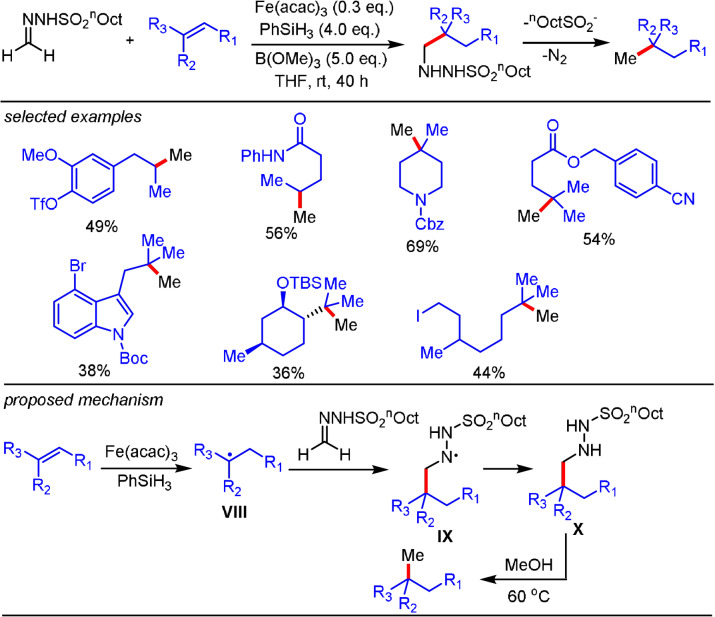
Iron‐catalyzed hydromethylation of alkenes.

In this context, Baran and co‐workers described that carbon radicals formed in the reaction of an intermediate [Fe‐H] species with an alkene partner can couple efficiently with formaldehyde‐derived *n*‐octylsulfonylhydrazone to give a hydromethylation product (Scheme 7).^[44]^ This reaction sequence could be initiated by using a stoichiometric or catalytic amount of Fe(acac)_3_, with excess B(OMe)_3_ being an essential additive. Alkyl hydrazide **X** was observed as the key intermediate, which decomposes upon heating to 60 °C in MeOH to eliminate sulfinate and nitrogen to yield the hydromethylated product, presumably by an ionic pathway.

In contrast to the organometallic‐functionalization method shown in Scheme 2 B, this radical‐based reaction tolerates base‐ and nucleophile‐sensitive functional groups, such as free alcohols, halides, azides, and esters. The iron catalytic system was further extended by Bradshaw and co‐workers to the alkylation of non‐activated alkenes by using *N*‐tosylhydrazones as radical acceptors.^[45]^

**Scheme 8:**
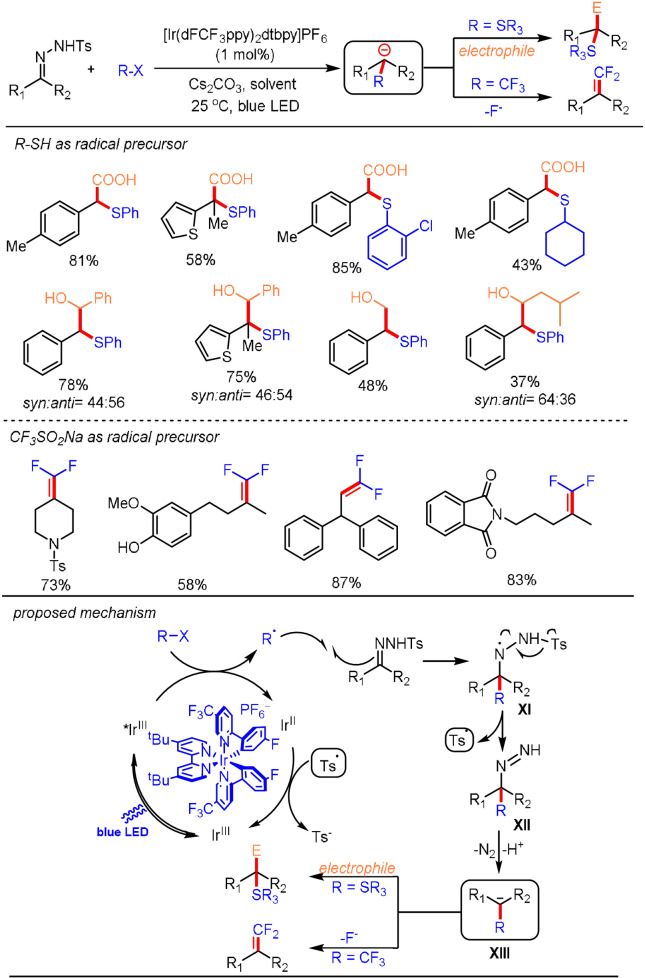
Photo‐WK reaction for the catalytic difunctionalization of sulfonylhydrazones.

Over the last few decades, photocatalysis has emerged as a powerful tool to access radicals for use in a broad range of chemical transformations through single‐electron transfer between the excited state of a photocatalyst and the substrates.^[46]^ In the context of our ongoing research on developing photoredox catalytic approaches for carbanion generation, we questioned if a photo‐Wolff–Kishner process could be realized by adding photogenerated radicals to sulfonyl hydrazones.^[47]^ The envisioned reaction process was initiated by single‐electron‐transfer oxidation of a suitable substrate by the excited photocatalyst to afford the radical and the reduced photocatalyst. Subsequent radical addition to the *N*‐sulfonylhydrazone produces an aminyl radical species **XI**, which undergoes β‐sulfonyl radical fragmentation to generate a functionalized diazene intermediate **XII**. The resulting diazene enters the WK process to give the key carbanion species **XIII**, which is capable of reacting with various electrophiles in a polar fashion. Finally, electron transfer between the reduced photocatalyst and the sulfonyl radical regenerates the catalyst, thus rendering the overall transformation redox‐neutral. When thiols were used as the radical precursors with an Ir‐based catalyst, the α‐substituted carbanions were efficiently produced, then trapped by CO_2_ or aldehydes. In the case of a CF_3_ radical, *gem*‐difluoroalkenes were produced as the final product after E1cB elimination of a fluoride anion from the resulting α‐CF_3_ carbanions (Scheme 8). This procedure shows that the merger of photocatalysis and a classical WK process leads to the efficient generation of a carbanion and broadens the available scope of electrophiles for carbanion trapping.

### 2.2 Generation of Carbanions by Catalytic Single‐Electron Reduction of Imines—Pathway B

**Scheme 9:**
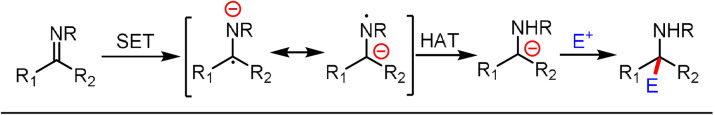
Photoredox catalytic generation of α‐amino carbanions—Fan/Walsh pathway.

The generation of imine radical anions through single‐electron reduction is well‐documented and has been employed for many transformations utilizing stoichiometric reductants.^[48]^ In recent years, new photocatalytic strategies have been developed as desirable alternatives for the single‐electron reduction of imine derivatives.^[10]^ Imine radical anions can show C‐centered and N‐centered radical reactivity depending on the precursor and the reaction conditions. The C‐centered radical species have been shown to engage in various radical coupling reactions,[^10^, ^11a^, ^11c^] while the N‐centered radical can abstract hydrogen atoms from the reaction medium to give an α‐amino carbanion. This intermediate is capable of reacting with various electrophiles in an ionic fashion, as pioneered by Fan and Walsh (Scheme 9).

**Scheme 10:**
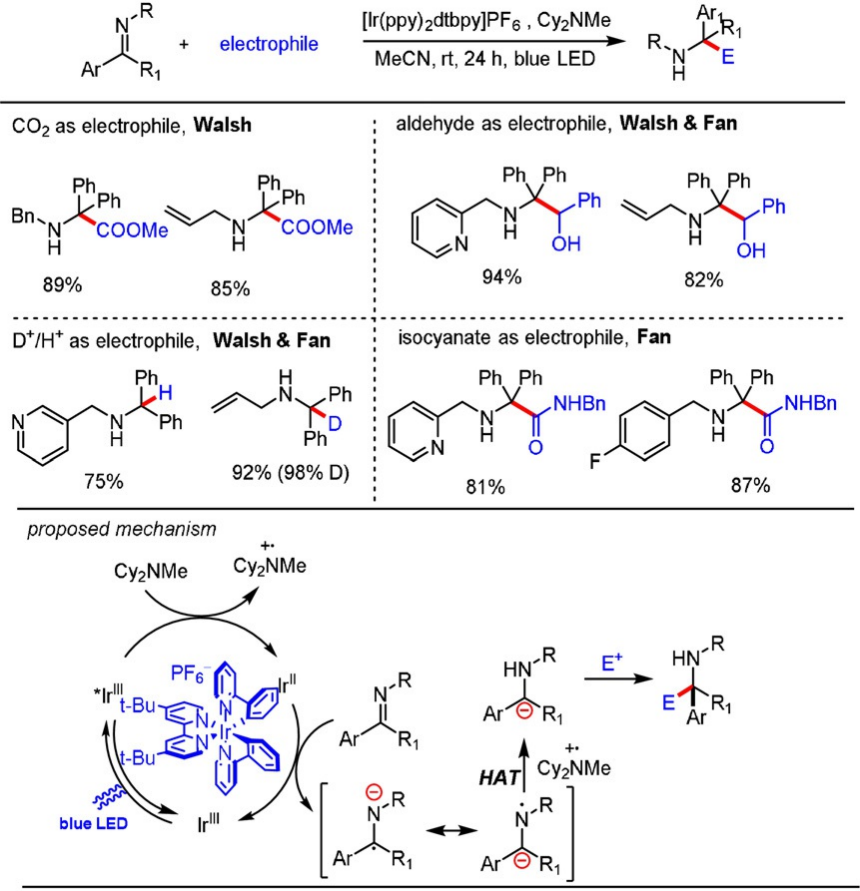
Photoredox catalytic system developed by Fan and Walsh for the generation of carbanions from imines.

In 2018, Fan, Walsh et al. reported a photoredox catalytic procedure for the hydrocarboxylation of *N*‐alkyl and *N*‐aryl imines with CO_2_ by using Ir(ppy)_2_(dtbbpy)PF_6_ as a photocatalyst and Cy_2_NMe as the terminal reductant.^[49]^ Their mechanistic proposal commences with the reductive quenching of the photoexcited Ir^3+^ species by Cy_2_NMe to generate the reducing Ir^2+^ species and the amine radical cation. Subsequent single‐electron transfer between the imine and the Ir^2+^ species gives an amine radical cation and regenerates Ir^3+^. The generated N‐centered radical anion reacts with the amine radical cation in a HAT process to provide the key carbanion intermediate, which was then trapped by CO_2_ to afford a carboxylic acid as the product after protonation. Furthermore, they found that the carbanions generated in the reaction are efficiently trapped by protons,^[50]^ aldehydes,^[51]^ or isocyanate^[52]^ (Scheme 10).

**Scheme 11:**
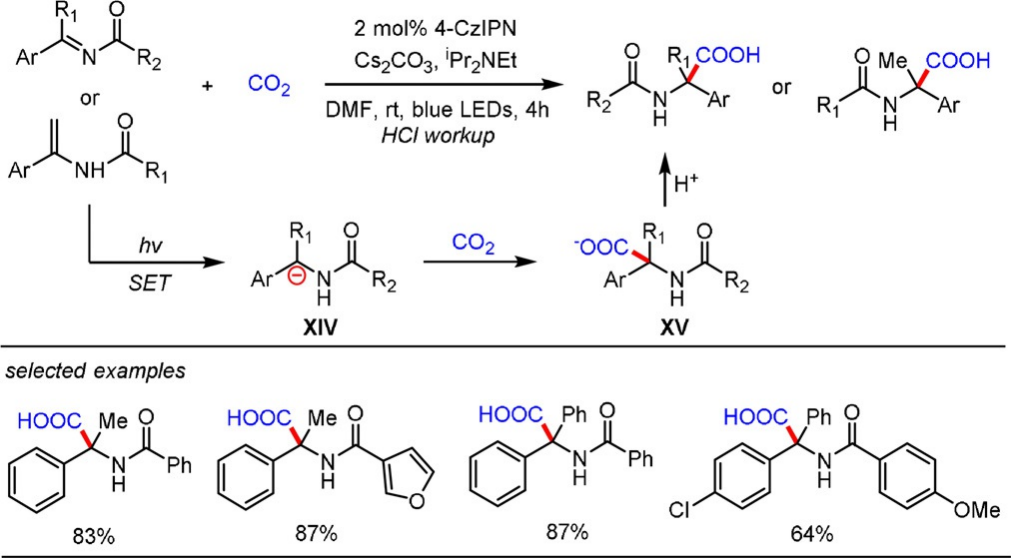
Photoredox catalytic hydrocarboxylation of imines developed by Yu and co‐workers.

A similar photoredox catalytic procedure for the hydrocarboxylation of enamides and aromatic imines with CO_2_ (1 atm) was independently discovered by Yu and co‐workers (Scheme 11).^[53]^ The reaction relies on the photochemical reduction of the imine using a tertiary amine (DIPEA) as a sacrificial reductant to generate the α‐amino carbanion **XIV** as the key intermediate for CO_2_ trapping. The authors conducted a series of control experiments and deuterium‐labeling studies to prove the existence of the carbanion intermediate in the reaction. However, a specific pathway for the generation of α‐amino carbanion **XIV** has not been provided. From a mechanistic point of view, this species could be produced through a SET/HAT mechanism as shown in Scheme 9 or a photocatalytic aza‐Bouveault‐Blanc reduction process, which will be discussed in Section 2.3.

### 2.3 Generation of Carbanions by Catalytic Sequential Single‐Electron Reduction of a Carbonyl Compound—Pathway C

**Scheme 12:**
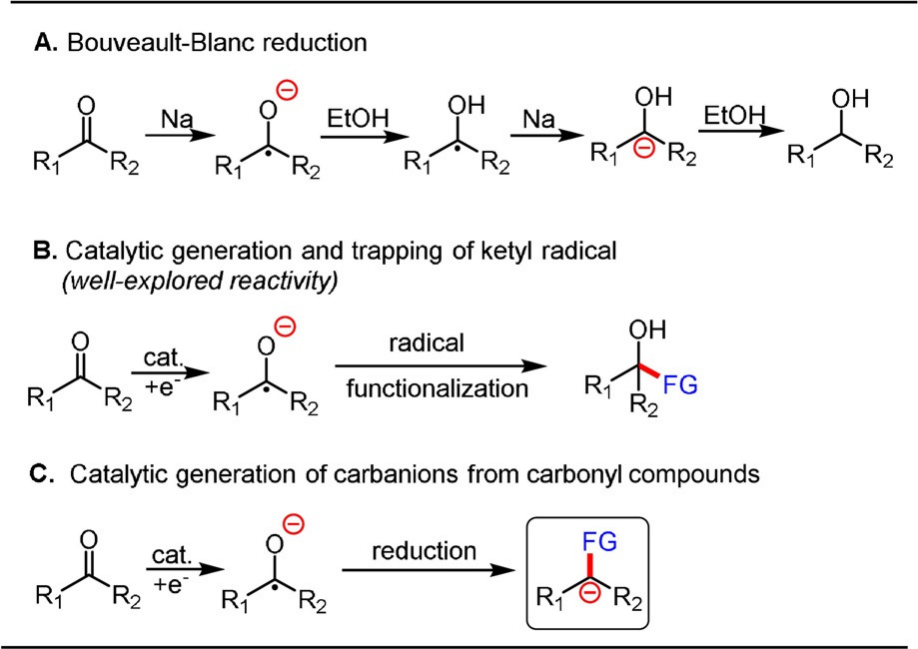
Generation of carbanions by the sequential reduction of carbonyl compounds.

Single‐electron reduction of carbonyl groups results in the formation of a ketyl radical, a reactive intermediate that can participate in a wide range of chemical transformations. Although the resonance structure of a ketyl radical has carbon radical and carbon anion character, the most explored reactions, such as radical–radical coupling and radical addition to double bonds, arise from its carbon radical nature.^[48c]^ In contrast to imine radical anions, the generation of a carbanion from a carbonyl group requires a second single‐electron reduction process of the ketyl radical. This pathway dates back to the well‐known Bouveault‐Blanc reduction, wherein an α‐hydroxy carbanion is produced as a key intermediate through two sequential single‐electron reduction steps of esters by sodium metal (Scheme 12 A).^[54]^ Classic methods to induce the carbonyl reduction rely on strong reductants such as alkali metals,[^8i^, ^55^] vanadium,^[8h]^ titanium,^[56]^ tin,^[57]^ zinc,^[58]^ and samarium diiodide (SmI_2_).[^48c^, ^59^] In this context, visible‐light‐induced photocatalysis has been developed as an ideal alternative to access ketyl radicals from carbonyl compounds by leveraging the reduction power of photocatalysis. As a consequence, a wide range of C−C bond‐coupling reactions have been discovered by reacting ketyl radicals with C‐centered radicals or unsaturated bonds (Scheme 12 B). The advances in exploiting the reactivity of ketyl radicals for C−C bond construction have been summarized in several recent reviews.^[11]^ We will, therefore, focus on the catalytic generation of carbanions by the sequential reduction of carbonyl compounds (Scheme 12 C).

**Scheme 13:**
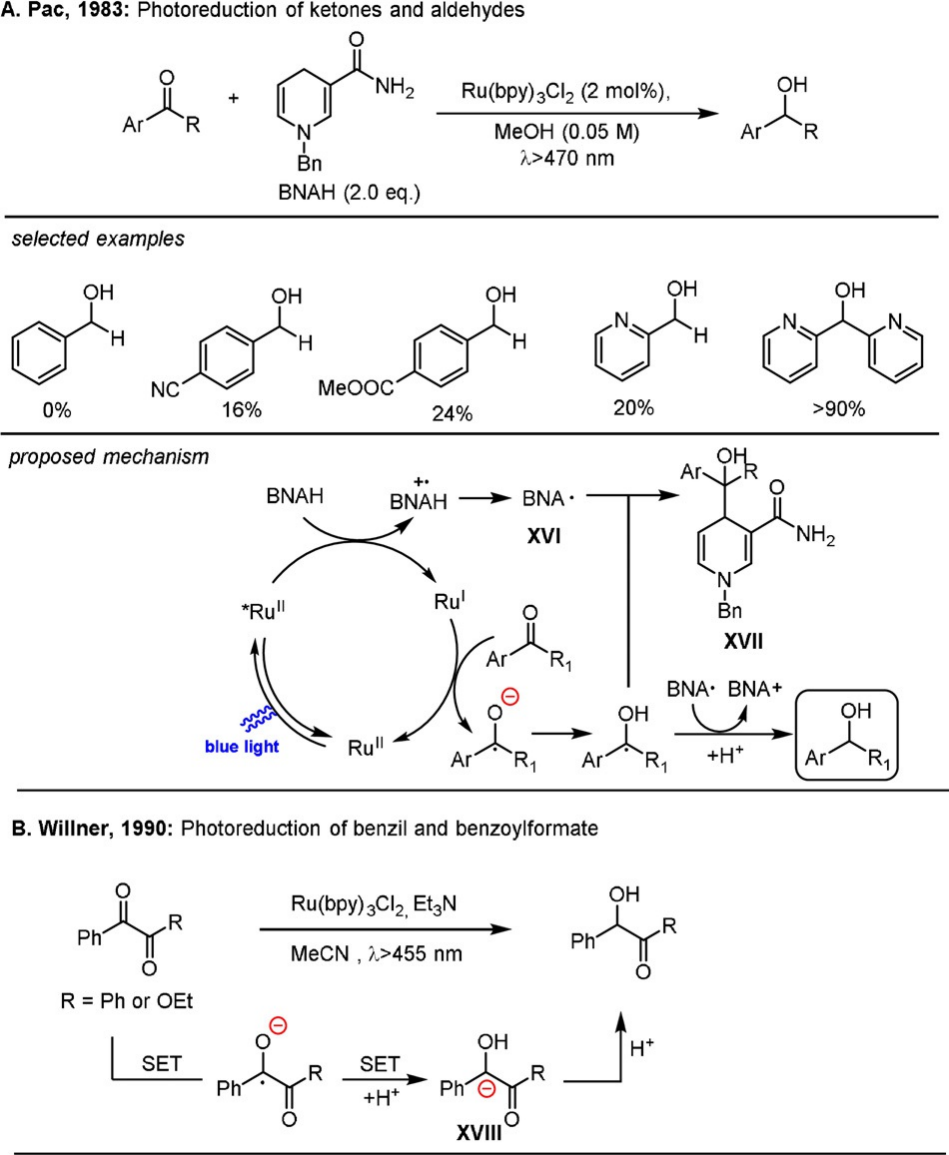
Photoinduced Ru‐catalytic generation of carbanions by the sequential reduction of carbonyl compounds.

In 1983, Pac and co‐workers reported the first example of a photoredox catalytic reduction of carbonyl compounds in the presence of Ru(bpy)_3_Cl_2_ as the photocatalyst and *N*‐benzyl‐1,4‐dihydronicotinamide (BNAH) as the reductant (Scheme 13 A).^[60]^ In the proposed mechanism, the excited state of *Ru(bpy)_3_^2+^ was quenched by BNAH to produce Ru(bpy)_3_^+^ and BNA^.^ (**XVI**) after deprotonation. Single‐electron transfer occurred between Ru(bpy)_3_^+^ and the carbonyl group to yield a ketyl radical and regenerate the photocatalyst. The resulting ketyl radical can undergo either a single‐electron transfer process with BNA^.^ to give an alcohol or radical–radical coupling with BNA^.^ to form compound **XVII**. The interaction between the ketyl radical and **XVI** was dependent on the electronic properties and structure of the carbonyl compounds, and it was found that the existence of electron‐withdrawing substituents and steric hindrance favors electron transfer between the two species. Later, a similar photoredox catalytic system for the reduction of activated ketones, including benzil and ethyl benzoylformate, was reported by Willner et al. with Ru(bpy)_3_Cl_2_ as the photocatalyst and Et_3_N as the reductant.^[61]^ The reaction involves two successive photoinduced SET processes to generate the α‐hydroxy carbanion **XVIII**, which was protonated to afford the hydrogenated product (Scheme 13 B).

**Scheme 14:**
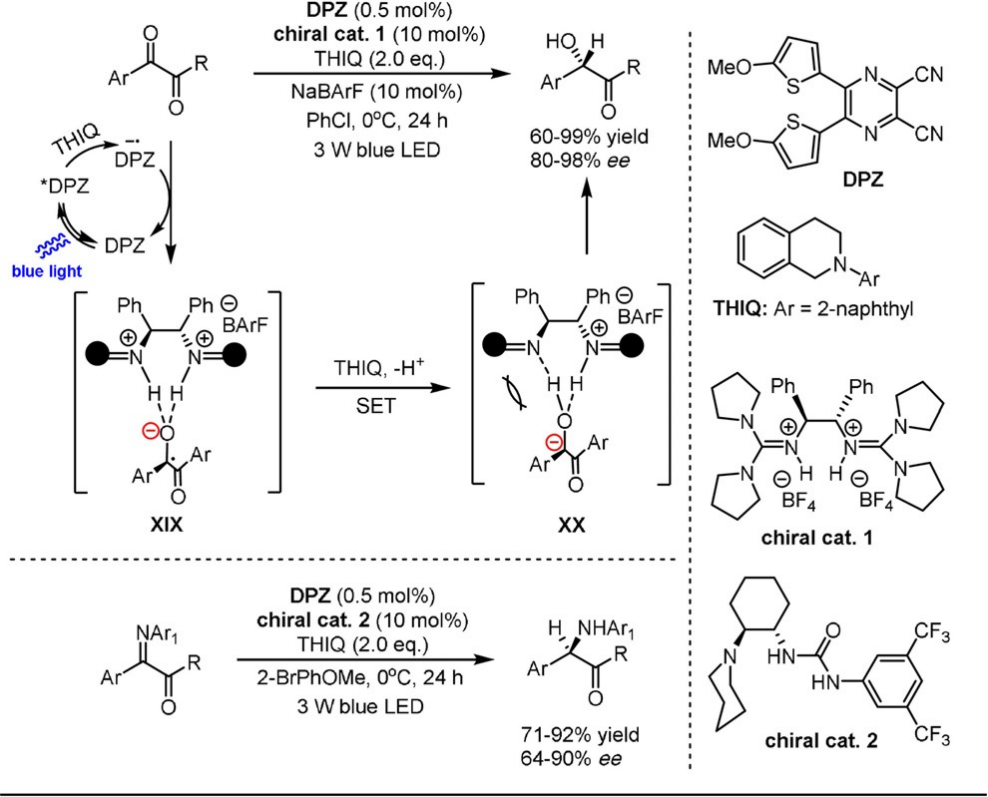
Photocatalytic enantioselective hydrogenation of 1,2‐diketones and α‐keto ketimines.

Recently, Jiang and co‐workers demonstrated a unique dual photo/asymmetric Brønsted acid catalysis system for the enantioselective hydrogenation of 1,2‐diketones (Scheme 14).^[62]^ The catalytic system consists of an organic photocatalyst (DPZ), a chiral guanidinium salt catalyst **1**, a borate additive NaBArF, and an electron donor *N*‐2‐naphthyl tetrahydroisoquinoline (THIQ). Noncovalent bonding between the Brønsted acid and the ketyl intermediate was found to be crucial for the reaction process. The second reduction of intermediate **XIX** was accomplished by THIQ to afford carbanion **XX**, which was poised for the subsequent enantioselective protonation process. The authors noted that the reaction occurs in the absence of DPZ through an electron‐donor‐acceptor mechanism, reaching comparable yields and enantioselectivities to those obtained in the catalytic photoredox system. The photocatalytic system was further applied for the asymmetric hydrogenation of α‐keto ketimines to produce α‐amino ketones by exchanging the chiral catalyst derived from guanidinium salt **1** with a chiral 1,2‐cyclohexanediamine‐based catalyst **2** containing urea and tertiary amine units.

**Scheme 15:**
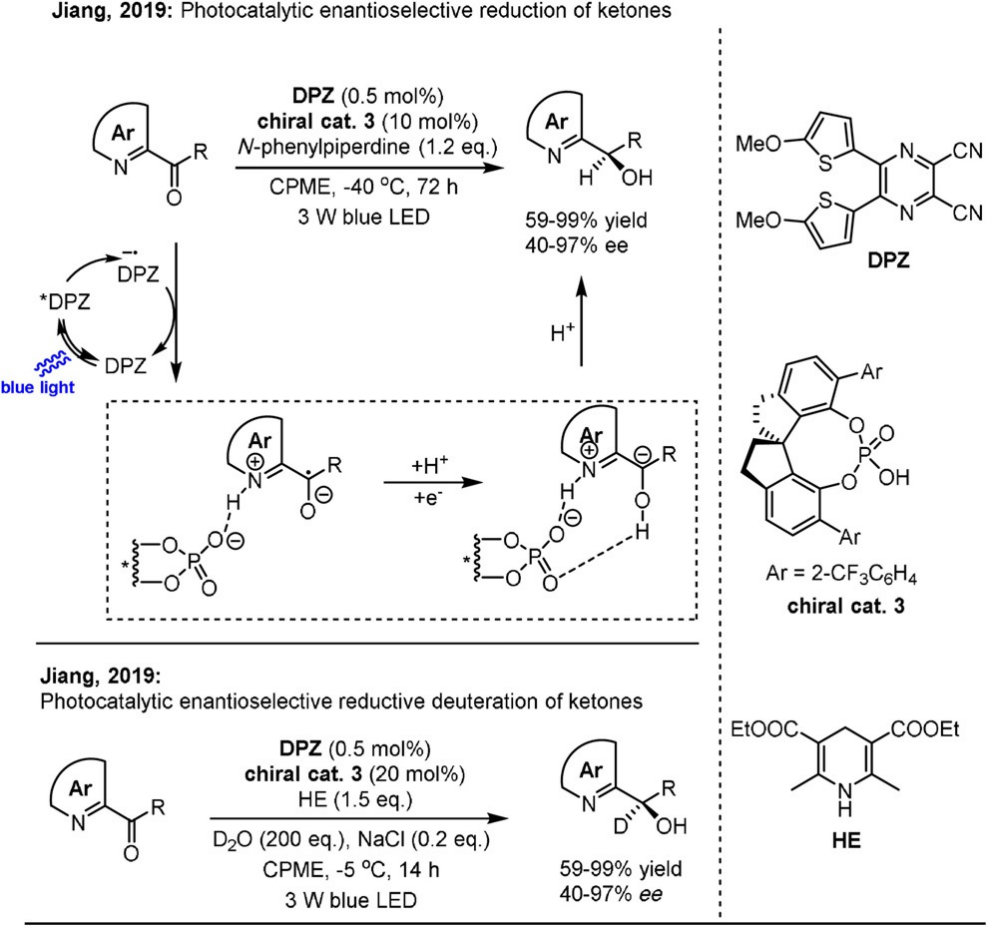
Photocatalytic enantioselective reduction of ketones.

Later, the same group extended this dual catalysis strategy to the asymmetric reduction of azaarene‐based ketones (Scheme 15).^[63]^ Similarly, ketones underwent two single‐electron reduction processes to give carbanions that participate in enantioselective protonation or deuteration when using SPINOL‐CPA **3** as a chiral catalyst. A wide range of chiral azaarene‐based secondary (deuterated) alcohols with diverse substitution patterns could be prepared in high yields with moderate to excellent enantioselectivity using this procedure.

**Scheme 16:**
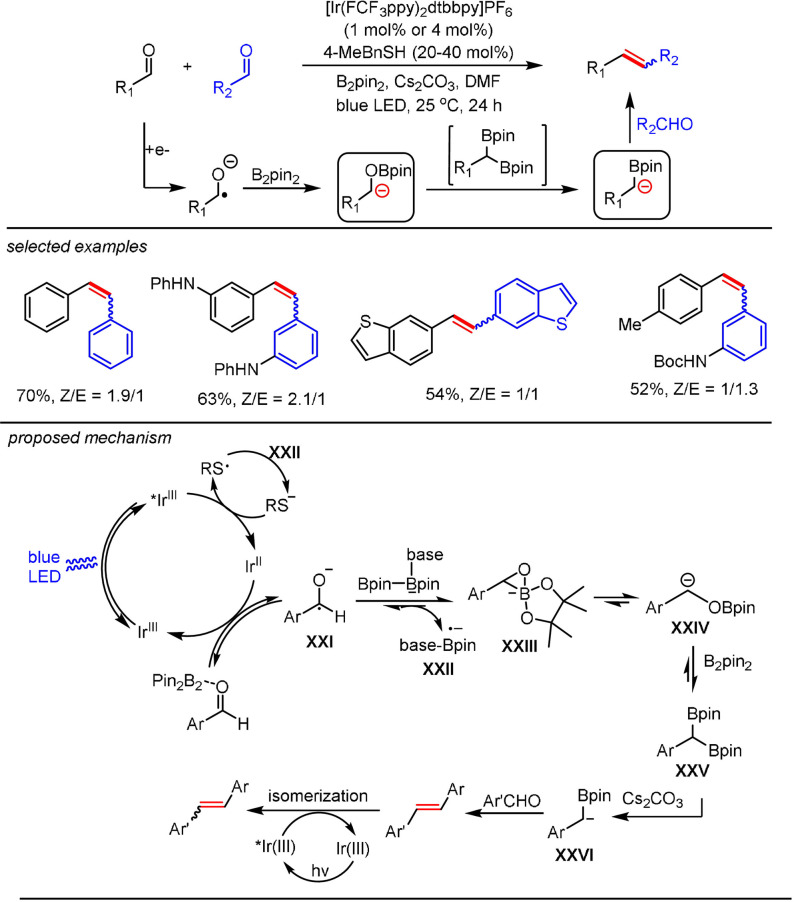
Photocatalytic generation of α‐boryl carbanions.

The aforementioned methods relied on the photocatalytic successive single‐electron reduction of an activated carbonyl functionality to access α‐hydroxy carbanions, thus enabling the facile hydrogenation of these ketones. We speculated that the ketyl radical from a photoinduced single‐electron reduction process could be further deoxygenated by an external reductant to afford other types of functionalized carbanions. In 2019, we demonstrated that a ketyl radical generated from an aromatic aldehyde by the photoredox catalytic system could be deoxygenated with B_2_pin_2_ to yield an α‐boryl carbanion (Scheme 16).^[64]^ The key to obtaining the desired reactivity was the use of a thiol as a co‐catalyst for shuttling electrons from B_2_pin_2_ to the photocatalytic system. The resulting α‐boryl carbanion reacted with another molecule of benzaldehyde to afford the alkene as the final product, thus providing a mild and efficient photocatalytic McMurry reaction. The reaction system was applicable to the synthesis of both symmetrical and unsymmetrical alkenes with broad substrate scope and a high level of functional‐group tolerance. Based on the in situ NMR studies and experimental results, we propose the following reaction mechanism: reductive quenching of the excited Ir^III^ complex by thiolate affords a thiyl radical and the reduced photocatalyst Ir^II^. The single‐electron transfer between the Ir^II^ species and benzaldehyde regenerates the photocatalyst and yields a ketyl radical **XXI**, which participates in a radical borylation‐“bora‐Brook” rearrangement sequence to give α‐oxyboryl carbanion **XXIV** and a base‐bound boryl radical anion **XXII**. Reduction of the thiyl radical by **XXII** regenerates the thiolate. The α‐oxyboryl carbanion **XXIV** reacted with another molecule of B_2_pin_2_ to yield the 1,1‐benzyldiboronate ester **XXV**, which underwent base‐assisted deborylation to give an α‐boryl carbanion **XXVI**. Subsequent B‐O elimination after nucleophilic addition to another aldehyde and an energy‐transfer process yielded a mixture of the *E*‐ and *Z*‐alkenes.

## 3 Conclusion and Outlook

We have summarized recent advances in the generation of carbanionic intermediates by catalytic carbonyl Umpolung strategies. Three reaction pathways yield carbanions starting from carbonyl compounds. First, the merger of the Wolff–Kishner reaction with catalysis provides efficient methods for the generation and/or trapping of carbanionic species, which impressively revitalizes this century‐old chemistry. Second, α‐amino carbanions are obtained by exploiting the carbanionic property of an imine radical anion arising from a catalytic single‐electron reduction process. Besides this, catalytic strategies have been applied for the production of carbanionic species by the successive reduction of carbonyl functionalities. In comparison to catalytic carbonyl Umpolung reactions involving an acyl anion, research addressing the analogous catalytic chemistry of alkyl carbanion equivalents from carbonyl compounds has received considerably less attention. One reason was the absence of suitable catalytic methods. This has changed and the combination of the classic reaction modes with recent transition‐metal or photocatalysis has demonstrated a great potential for the generation of alkyl carbanions that are capable of participating in various C−C bond‐forging reactions in a mild and controllable manner.

However, despite the advances in this field, further development is still required to address the limitations and challenges. The catalytic radical‐initiated WK reaction using a sulfonyl hydrazone as a radical acceptor has appeared in only a limited number of literature reports (Scheme 1, pathway A). We anticipate that synthetic applications of this chemistry can be further explored towards addressing the following aspects: 1) Expanding the scope of radical precursors for the production of carbanions, 2) exploiting the reactivity of the generated diazene intermediates for more transformations (e.g. pericyclic arrangements to construct unsaturated bonds), and 3) exploring the possibility of combining other types of catalysis (for radical generation) with the WK process to produce anionic species. In the case of strategies involving the catalytic single‐electron reduction of imines and carbonyl compounds (Scheme 1, pathways B and C), related transformations remain in their infancy and suffer from a limited substrate scope as well as a low diversity of trapping electrophiles. Efforts are, therefore, expected to be devoted to the development of more efficient catalytic systems to address these challenges and limitations.

In our opinion, numerous exciting opportunities in catalytic carbonyl Umpolung for carbanion generation still wait to be discovered, thereby expanding the arsenal of valuable transformations in organic synthesis. We hope this Minireview will stimulate more chemists to exploit new catalytic methods and strategies to address the above‐described challenges in the future.

## Conflict of interest

The authors declare no conflict of interest.

## Biographical Information

*Shun Wang received his master's degree in organic chemistry in 2016 at Xi′an Jiaotong University (Xi′an, China) under the supervision of Prof. Li‐Na Guo and Prof. Xin‐Hua Duan. He then joined the group of Prof. Burkhard König at the University of Regensburg (Regensburg, Germany) as a PhD student. He completed his PhD in 2021. His research interests focus on the photoredox catalytic generation of carbanions through carbonyl Umpolung and organic anion photocatalysis*.

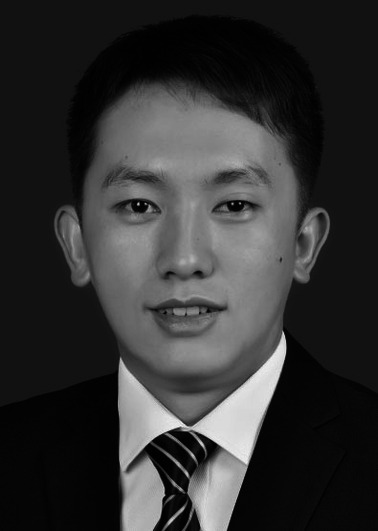

## Biographical Information

*Burkhard König received his PhD in 1991 from the University of Hamburg, Germany. He then carried out postdoctoral research with Prof. M. A. Bennett, Research School of Chemistry, Australian National University, Canberra, and Prof. B. M. Trost, Stanford University. Since 1999, he has been a full professor of organic chemistry at the University of Regensburg, Germany. His research focuses on the photocatalytic generation of ionic reactive intermediates and control of substrate–photocatalyst interactions to enhance reaction selectivity*.